# microRNA Prognostic Signature for Postoperative Success of Metastatic Orthopedic Cancers: Implications for Precision Microsurgery

**DOI:** 10.3389/fcell.2021.704505

**Published:** 2021-07-02

**Authors:** Shi-Bao Xu, Rong-Hao Fan, Xiao Qin, Rui-Ming Han

**Affiliations:** Department of Orthopedics, JiaoZuo People’s Hospital, Jiaozuo, China

**Keywords:** bone metastasis, miR-203, precision medicine, miR-10b, post-operative

## Abstract

The importance of miRNA prognostic signature in cancer, particular cancer metastasis is increasingly being realized. Bone metastasis from several primary human cancers can be managed in clinics by surgical intervention but the prognostic impact of miRNA signature on post-surgery outcome of patients is unknown. This study evaluated a miRNA signature for post-operative outcome of patients with bone metastatic disease. First, the miRNAs, miR-135, miR-203, miR-10b, miR-194, miR-886, and miR-124 were evaluated in bone metastatic tissues, relative to adjacent control tissue. The cohorts of samples (*n* = 44) consisted of bone metastatic cancer patients with primary lung (*n* = 18) or breast cancer (*n* = 26). miR-203 was significantly down-regulated while miR-10b was significantly up-regulated in bone metastasis. Additionally, miR-135 was significantly differentially expressed in the primary lung cancer patients while miR-194 in primary breast cancer patients. The low miR-203- high miR-10b expression was designated high risk group and, compared to the low risk group (high miR-203-low miR-10b expression). Patients with the signature high risk fared significantly better with surgical intervention, in terms of survival at 12 months time point (40% survival with surgery vs. 10% survival without surgery), as revealed by retrospective analysis of patient data. This work reveals potential utilization of miRNA expression levels in not only the general prognosis of cancer metastasis but also the prognosis of surgical intervention with implication for better stratification of patients.

## Introduction

Primary cancers from other organs metastasize to bones very frequently, with some evidence that metastasis to bones are third most common metastasis, ranked just behind metastasis to lungs and livers (Bellido and Plotkin, 2011; Sevimli and Korkmaz, 2018). This represents a major clinical issue as metastasis to bones are commonly presented in the clinics than the primary bone tumors (Macedo et al., 2017; Sevimli and Korkmaz, 2018; Jayarangaiah and Kariyanna, 2021). Tumor cells from any primary site can metastasize to bones but the most frequently reported primary sites from where tumors metastasize to bones are lung, breast, prostate, kidney, and thyroid (Maccauro et al., 2011; Macedo et al., 2017).

Bone metastasis are generally lethal particularly because of their typical late stage diagnosis (Cetin et al., 2015). Some treatment options are available for the patients and these mainly include radiation therapy or surgical interventions. Surgery is often performed with the aim of improving quality of life. Surgery is needed to stabilize fractured or vulnerable bones. Surgery also relieves spinal cord compression that can have many secondary manifestations such as numbness, difficulty walking or using arms, loss of bowel or bladder control, and even paralysis. While the clear numbers on the lethality of bone metastatic cancers, particularly post-surgery, are not available, it has been estimated that 30 day post-surgery mortality rate could be as high as 7.1% (Gallaway et al., 2020). Furthermore, the progression of disease varies in individual patients making it important to understand the underlying genetic or epigenetic factors predisposing patients to advanced disease.

Epigenetic changes, particularly dysregulated expression levels of miRNAs, can be of prognostic importance (Ahadi, 2020; Chen et al., 2020; Lv et al., 2020) and, therefore, this study was conducted to evaluate such potential of miRNAs, especially with regards to the success of surgery of metastatic bone cancers. Expression of select miRNAs was first analyzed in the bone metastasis of 44 patients with bone metastasis, relative to the adjacent bone material and then I applied this information to evaluate the impact of surgery on outcome of patients by diving the patients into a high risk vs. low risk group based on the miRNA signature.

## Materials and Methods

### Patients Information

Patient data was extracted from the patients enrolled at JiaoZuo People’s Hospital between January 2014 and December 2020. The study was conducted after approval from the Ethics Committee at the JiaoZuo People’s Hospital (Approval Number 18/1334). Informed consent was obtained from all patients prior to the collection of samples. Two patient cohorts were selected for the study with a total of 44 patients. One cohort consisted of patients with primary lung cancer with bone metastasis (*n* = 18) and the other cohort consisted of patients with primary breast cancer with bone metastasis (*n* = 26). The lung cancer cohort consisted of 11 males and seven females. All this information is provided in Table 1.

**TABLE 1:** Patient data.

Tumor primary site	Number of patients (Total *n* = 44)	Females	Males
Lung	18	7 (38.9%)	11 (61.1)
Breast	26	26 (100%)	0 (0%)

### RNA Isolation

Biopsy tissues were immediately froze upon collection. At the time of analysis, they were thawed and homogenized with mechanical force using metal bead agitation at 4 °C (Next Advance Bullet Blender^®^ Storm with Navy 5 mL Lysis Kit). High-quality total RNA was purified from bone tissues lysed with TRIzol^®^ using Direct-zol^TM^ RNA MiniPrep (Zymo Research), as per vendor’s protocol and as detailed by other researchers (Cho et al., 2016). The quality of RNA was tested using a Nanodrop 2000 instrument (ThermoFisher Scientific, China) and Bioanalyzer (Agilent Technologies, Japan).

### qRT-PCR and miRNA Analysis

Primers and detection reagents from Qiagen (China) were used to detect miRNAs in patient samples, as described by other researchers (Pan et al., 2020). Commercially available RNAs-free water was used throughout the analysis. RT^2^ First Strand Kit (Qiagen, China) was employed to synthesize first strand of cDNA using 1 μg RNA, to which 2 μl of genomic DNA elimination mix was added, mixed and incubated 10 min at 42°C, followed by immediate transfer to ice for 1 min. Reverse transcription mix, consisting of 5× buffer and Reverse Transcriptase, was then prepared, as per vendors protocol and added to RNA. Further incubation was for 15 min at 42°C. Thereafter, the reaction was halted by 5 min incubation at 95°C.

### Statistical Methods

All experiments were independently performed at least three times with triplicate repeats. The data was analyzed by a biostatistician to whom the identity of samples was not revealed. To evaluate if two datasets were significantly different, a *p* value was calculated using Student *t* test or one way ANOVA assuming equal variables and two-tailed distribution (Pan et al., 2020). Prior to the statistical tests, datasets were log-transformed to ensure normal distribution. Only the *p* values ≤0.05 were considered to represent statistically significant miRNA expression levels.

## Results

### microRNAs Differentially Expressed in Bone Metastasis

**FIGURE 1:**
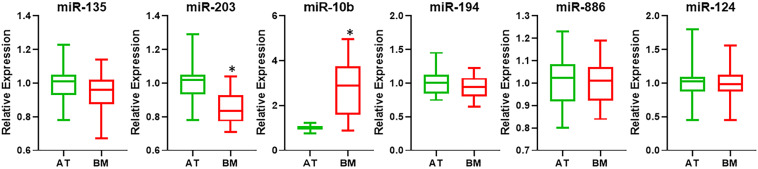
Differential expression of miRNAs. miRNAs were evaluated by qRT-PCR for their expression levels in bone metastatic tissue (BM), relative to their expression in the adjacent control tissue (AT). ^∗^*p* < 0.01.

I began the investigation by surveying the literature for miRNAs that could potentially serve as biomarkers for bone metastatic cancers. Based on the samples which consisted of bone metastasis from primary lung and breast cancers (Table 1), I focused on miRNAs that were earlier reported to be of importance in this context. I chose miR-135, miR-203, miR-10b, miR-194, miR-886, and miR-124 because of the reports on their ability to play a role in bone metastasis from either primary lung cancer or breast cancer (Cao et al., 2013; Wu et al., 2014; Taipaleenmäki et al., 2015; Yoo et al., 2017; Cai et al., 2018). When analyzed for expression in bone metastasis, relative to adjacent non-cancerous tissues, I found significantly down-regulated miR-203 and significantly up-regulated miR-10b (Figure 1) with *p* < 0.01. The other miRNAs tested (miR-135, miR-194, miR-886, and miR-124) were not found to be differentially expressed.

### Differentially Expressed miRNAs in Lung Cancer Bone Metastasis

**FIGURE 2:**
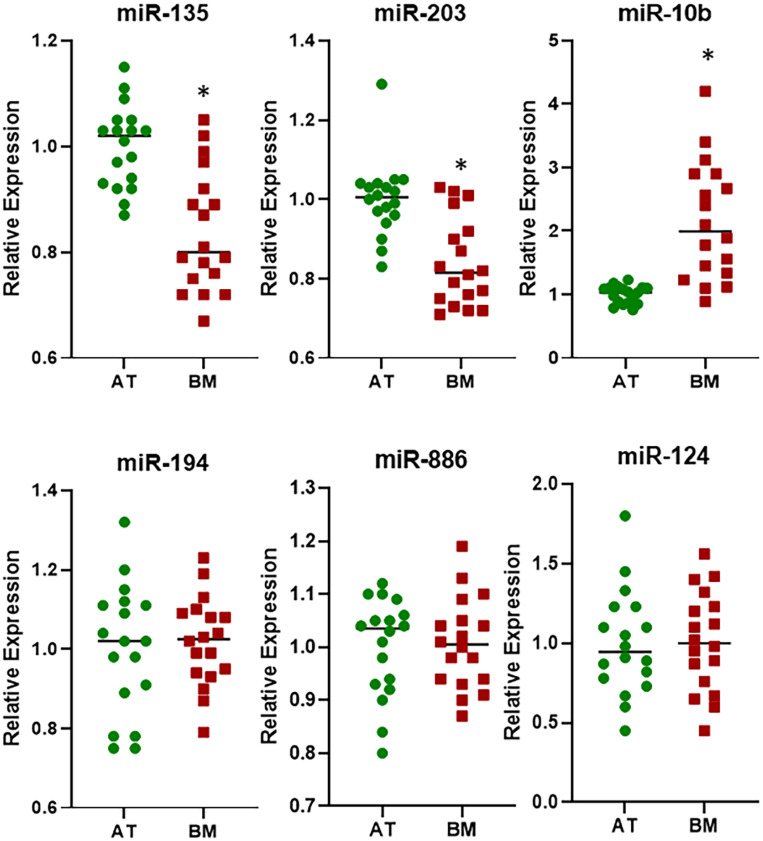
Differential expression of miRNAs in lung cancer cohort. miRNAs were evaluated by qRT-PCR for their expression levels in BM from patients with primary lung cancer, relative to their expression in the AT. ^∗^*p* < 0.01.

Since the patient cohort consisted of bone metastasis from primary lung and breast cancers, I next looked at the differential expression of the six miRNAs in individual samples to evaluate a possible role in metastasis from specific tissues. I first evaluated samples from patients who reported bone metastasis from primary lung cancers. As shown in Figure 2, now I observed three miRNAs (miR-135, miR-203, and miR-10b) to be significantly differentially expressed (*p* < 0.01) in the bone metastasis. This was interesting because miR-135 did not turn up significant values when checked in the pooled samples above. This suggests its possible role as a biomarker in bone metastasis from primary lung cancers. The rest three miRNAs (miR-194, miR-886, and miR-124) were still found to be expressed at almost same levels in bone metastasis as well as adjacent tissues.

### Differentially Expressed miRNAs in Breast Cancer Bone Metastasis

**FIGURE 3:**
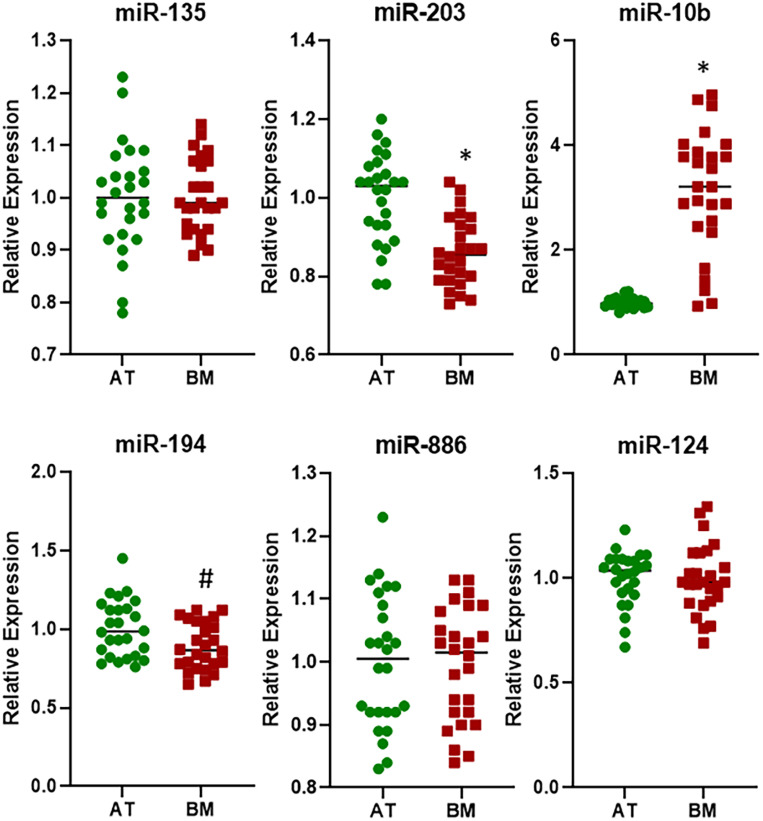
Differential expression of miRNAs in breast cancer cohort. miRNAs were evaluated by qRT-PCR for their expression levels in BM from patients with primary breast cancer, relative to their expression in the AT. ^∗^*p* < 0.01; ^#^*p* < 0.05.

Next, I focused on the samples that were bone metastasis from primary breast cancers. An analysis of 26 such samples for the expression of six miRNAs revealed that whereas the expressions of miR-135, miR-886, and miR-124 were not significantly different in bone metastasis, relative to adjacent tissue, the expressions of miR-203, miR-10b, and miR-194 were (Figure 3). While miR-203 and miR-10b returned significant values with *p* < 0.01, miR-194 was significantly differentially expressed with *p* < 0.05. This was another interesting observation as miR-194 was not found to be differentially expressed in pooled samples above.

### Gender Specific Differentially Expressed miRNAs in Lung Cancer Bone Metastasis

**FIGURE 4:**
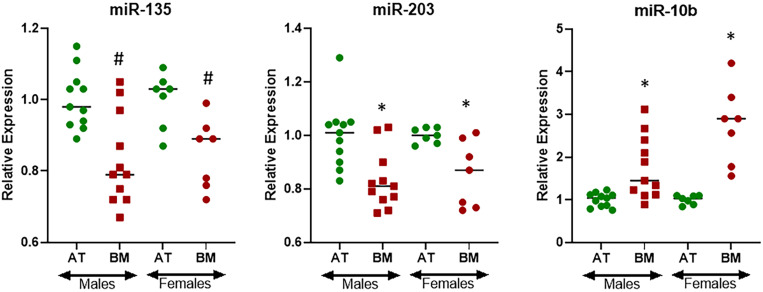
Gender-specific differential expression of miRNAs in lung cancer cohort. miRNAs were evaluated by qRT-PCR for their expression levels in BM from patients with primary lung cancer further stratified by gender, relative to their expression in the AT. ^∗^*p* < 0.01; ^#^*p* < 0.05.

The cohort of patients representing bone metastasis from primary lung cancers consisted of both males (61.1%) and females (38.9%) (Table 1) and therefore I asked the question if there could be gender specific differences in the expression of miRNAs. For this evaluation, I only focused on three miRNAs that were found to be differentially expressed at statistically significant levels in bone metastasis from primary lung cancers, i.e., miR-135, miR-203, and miR-10b. miR-135 was found to be expressed differentially in males and females with *p* < 0.05 while miR-203 and miR-10b were found to be expressed differentially in males and females with *p* < 0.01 (Figure 4). Interestingly, miR-135 seemed to be more differentially expressed in males while miR-10b seemed to be relatively more differentially expressed in females.

### miRNA Signature and Impact on Post-surgical Outcome

In the analyses thus far, I found two miRNAs (miR-203 and miR-10b) that were consistently differentially expressed in bone metastasis from primary lung and primary breast cancers. These two miRNAs could be the biomarkers for aggressive disease with poor outcomes. This could be of significance with regards to the post-surgery outcomes as well. Therefore, I next grouped all the patients in the cohort according to their status of miR-203 and miR-10b expression, for this retrospective analysis. The patients with low miR-203 and concomitant high miR-10b were presumed to be a high risk group while the patients with high miR-203 and low miR-10b were presumed to be the low risk group. Based on the available records on the survival of these patients, I analyzed whether surgical intervention in these groups impacted the overall survival. As revealed by statistical analyses (Table 2), the high risk group had poor overall survival compared to the low risk group. Interestingly, surgical intervention improved the survival in both groups. In the low risk group, surgical intervention increased survival at 12 months from 70 to 80% while in the high risk group, the effect of surgical intervention was even more significant and the survival improved from 10 to 40% at 12 months time point.

**TABLE 2:** Impact of surgical intervention on disease outcome, based on miRNA signature.

Group	Primary	6 months survival	12 months survival
miR-203-Low miR-10b-High (high risk)	Surgery	8/10 (80%)	4/10 (40%)
	No surgery	7/10 (70%)	1/10 (10%)
miR-203-High miR-10b-Low (low risk)	Surgery	9/10 (90%)	8/10 (80%)
	No surgery	9/10 (90%)	7/10 (70%)

*miR-203-Low/miR-10b-High: Patients with low expression of miR-135 and miR-203 and high expression of miR-10b in bone metastasis prior to surgery; miR-203-High/miR-10b-Low: Patients with high expression of miR-135 and miR-203 and low expression of miR-10b in bone metastasis prior to surgery.*

## Discussion

The value of miRNAs as biomarkers and possible targets of therapy with respect to metastatic cancers has been realized in many studies (Croset et al., 2015; Zhao et al., 2015). Thus, this study was designed using clinical samples to focus on the prognostic relevance of differentially expressed miRNAs in bone metastatic cancers. The cohort of samples consisted of bone metastasis from primary lung and breast cancers. These represent two of the very common primary sites from which tumors metastasize to bones (Al Husaini et al., 2009; Pulido et al., 2017; Guerrieri et al., 2020).

Firstly pooled samples were analyzed, i.e., samples representing bone metastasis from primary lung as well as breast cancers. This was followed by analyses in specific cohorts, i.e., samples representing bone metastasis from either primary lung cancers or the breast cancers. This led to an interesting observation that one miRNA (miR-194) was differentially expressed in the breast cancer cohort (and not in the lung cancer cohort) while a different miRNA (miR-135) was differentially expressed in the lung cancer cohort (and not in the breast cancer cohort). This justified the analyses in specific cohorts as the pooled samples did not return significant values. Also, this suggests that these two miRNAs might play a role in metastasis from specific primary sites and future studies should focus on this aspect of these two miRNAs.

Even though miR-135 was found to be differentially expressed in bone metastasis from primary lung cancer in this study, it is interesting that this miRNA has actually been shown to play a role in bone metastasis from primary breast cancer (Taipaleenmäki et al., 2015). On the other hand, miR-194, which we report here to be differentially expressed only in bone metastasis from primary breast cancers (and not from the primary lung cancers) has actually been reported to be involved in bone metastasis from lung cancers (Wu et al., 2014). Thus, these observations provide novel information about the possible role of these miRNAs. More importantly, this points to the lack of clear information available with regards to the role of specific miRNAs in cancer metastasis. This is further supported by the observation with the two miRNAs (miR-886 and miR-124) which were never found to be statistically differentially expressed even though they were chosen, like the other four miRNAs, based on the published literature. For miR-886, its reported role in bone metastasis has been recorded from primary small cell lung carcinoma (Cao et al., 2013) while the patients in current cohort represented non-small cell lung carcinoma, the more common form of lung cancer (Molina et al., 2008), and this could be the reason for discrepancy.

In addition to cancer specific cohort analysis, gender specific analysis in the lung cancer cohort was also performed. This was considered to be of relevance as breast cancer cohort consisted of 100% females. This approach was clearly justified as some differences in differential expression of miRNAs were observed between the genders. For example, miR-10b was more highly expressed in bone metastasis of female lung cancer patients. Interestingly, miR-10b was also found to be the most differentially expressed miRNA in breast cancer cohort. Similarly, the results also revealed gender specific differential expression of miR-135. It was relatively more differentially expressed in males from lung cancer cohort. Interestingly, it was not significantly different in breast cancer cohort and that’s why possibly did not return significant values in overall pooled samples.

Based on the overall analyses, the two miRNAs that stood out as possible biomarkers for bone metastatic cancers were miR-10b and miR-203. While miR-203 was down-regulated in bone metastasis, miR-10b was up-regulated. The results support the published tumor suppressive role of miR-203 (Deng et al., 2016; Zhou et al., 2017) and the metastasis-inducing activity of miR-10b (Ma et al., 2007; Ahmad et al., 2014). I, therefore, focused on these two miRNAs for further evaluation even though two miRNAs, i.e., miR-135 and miR-194 seemed to be of interest in individual cohorts.

miRNAs and their role in precision medicine, particularly with respect to bone metastasis, is increasingly being appreciated (Zoni and van der Pluijm, 2016; Wood and Brown, 2020). The overarching goal of this study was to evaluate if the miRNA signature can help predict the post-surgical outcome of patients with bone metastasis. There is no information on the subject and a miRNA signature consisting of tumor suppressive miR-203 and the oncogenic miR-10b was evaluated. The combined expression of this miRNA signature indeed seemed to suggest a clear advantage of surgical intervention in patients. Thus, the benefits of surgery can go much beyond the known benefits involving quality of life. The miRNA signature can help predict the patient outcome and the information such as this can go a long way in furthering precision medicine, particularly precision microsurgery, on which there is almost complete lack of data.

## Data Availability Statement

The original contributions presented in the study are included in the article/supplementary material, further inquiries can be directed to the corresponding author.

## Ethics Statement

The studies involving human participants were reviewed and approved by Ethics Committee at the JiaoZuo People’s Hospital. The patients/participants provided their written informed consent to participate in this study.

## Author Contributions

S-BX conceptualized the project, interpreted data, and drafted manuscript. S-BX, R-HF, XQ, and R-MH performed experiments. R-HF and R-MH analyzed data. XQ performed statistical analysis. S-BX, R-HF, XQ, and R-MH edited manuscript and approved submission.

## Conflict of Interest

The authors declare that the research was conducted in the absence of any commercial or financial relationships that could be construed as a potential conflict of interest.
